# How Much Does Plant Food (Fruits and Vegetables) Intake Affect the Prevention of Periodontal Disease in the Korean Elderly?

**DOI:** 10.3390/nu14214589

**Published:** 2022-11-01

**Authors:** Yongseok Kwon, Sohye Kim

**Affiliations:** 1 National Institute of Agricultural Sciences, 166 Nongsaengmyeong-ro, Wanju 55365, Korea; 2 Department of Medical Nutrition, Graduate School of East-West Medical Science, Kyung Hee University, Yongin 17104, Korea; 3 Nutrition Care Services, Seoul National University of Bundang Hospital, Seongnam 13620, Korea

**Keywords:** plant food, periodontitis, elderly, vegetable, fruits

## Abstract

This study, as part of a study on the dietary lives of elderly people, investigates the association between plant food (fruit + non-starchy vegetable) intake and the prevention of periodontal disease among elderly people aged over 65 years. A total of 4514 subjects over 65 years of age participated in a dental survey, health behavior interview, and 24 h dietary recall test for the Korea National Health and Nutrition Examination Survey (KNHANES). Subjects with energy intake of less than 500 kcal or more than 5000 kcal were excluded. The results showed that plant food intake was inversely associated with the prevalence of periodontal disease in the elderly Korean population. The prevalence of periodontal disease decreased with increasing plant food consumption. Compared with those in tertile 1 (T1), subjects in tertile 3 (T3) showed a decrease in periodontal disease of about 26.7% (OR = 0.733). In conclusion, the consumption of plant food lowers the risk of periodontal disease, suggesting that it should be encouraged among elderly Koreans.

## 1. Introduction

Periodontal disease (PD) is characterized by microbial-associated host-mediated inflammation and is a major risk factor for tooth loss, as it reduces alveolar bone density and damages the bone structure due to plaque on the dental surface [1,2,3,4]. The progression of PD is not only limited to the oral cavity but also affects diabetes [5,6], hypertension [7], osteoporosis, cardiovascular disease, stroke, chronic diseases, and mortality [1,8].

It is reported that 85% of the adult population over the age of 35 has PD [9], and according to the Global Burden Disease Study in 2015, the prevalence of PD increased by 25.4% between 2005 and 2015 [10]. Furthermore, according to the Korean National Health and Nutrition Examination Survey (KNHANES) result published by the South Korean Disease Control and Prevention Agency, there were more than 29.8% and 47.2% increases in PD in South Korean adults over 19 and 50, respectively [11]. The rapidly aging population in South Korea is highly likely to display an increase in PD [12,13,14]. Therefore, early detection and continuous preventative management after the treatment of PD are necessary [15].

Despite the lack of previous studies on PD mechanisms, it has been reported that nutrition intake is significant in maintaining healthy teeth and oral tissues and is closely related to oral health [16]. It is known that certain nutrients such as calcium [17], vitamin C [18], vitamin D [19], and immune-related proteins intakes [20] are correlated with PD. After analyzing the relationship between food groups and PD using KNHANES data, one study reported that there are different PD risks according to food groups [15]. Furthermore, in another recent study, it was shown that dairy products and fruit consumption are decreasing the prevalence of periodontitis [21,22]. Consequently, the consumption of various nutrients and food groups is reported to be affecting PD.

Various domestic and international studies have shown the relationships between the consumption of various food groups and nutrients and PD; however, the association in the elderly has not yet been investigated. Therefore, as South Korea is becoming an aged society, this study examined the correlation between diet and oral health in the elderly population. In particular, the prevalence of PD in people over 65 and its relationship with plant-based food (fruits and vegetables) were investigated. This study can contribute to the prevention of PD in elderly people and may provide basic data for dietary educational programs and policy content to improve older people’s dietary habits in preparation for a super-aged society.

## 2. Materials and Methods

### 2.1. Research Data

This study was based on the fifth and sixth Korea National Health and Nutrition Examination Survey (KNHANES) conducted in 2012 and 2015. The KNHANES is a nationwide, population-based, cross-sectional study that aims to assess the health and nutrition status of the Korean civilian, noninstitutionalized population. The KNHANES administered by the Korean Ministry of Health and Welfare and a stratified multistage probability design were used with subject selection from sampling units using household registries. It also provides basic data for framing a health policy such as for improving the nutrition of the people, preventing diseases, and developing health promotion programs [23]. The sample survey plots are extracted from the KNHANES, and the study was conducted as a year-round survey between January and December [24]. In addition, the KNHANES consists of health interviews, health examinations, and 81 nutritional surveys, among which the nutritional survey aims to understand the food and nutritional intake and eating habits of Koreans: a food frequency questionnaire, dietary life, and food intake are recorded according to a 24 h recall method [23].

### 2.2. Subjects

**Figure 1:**
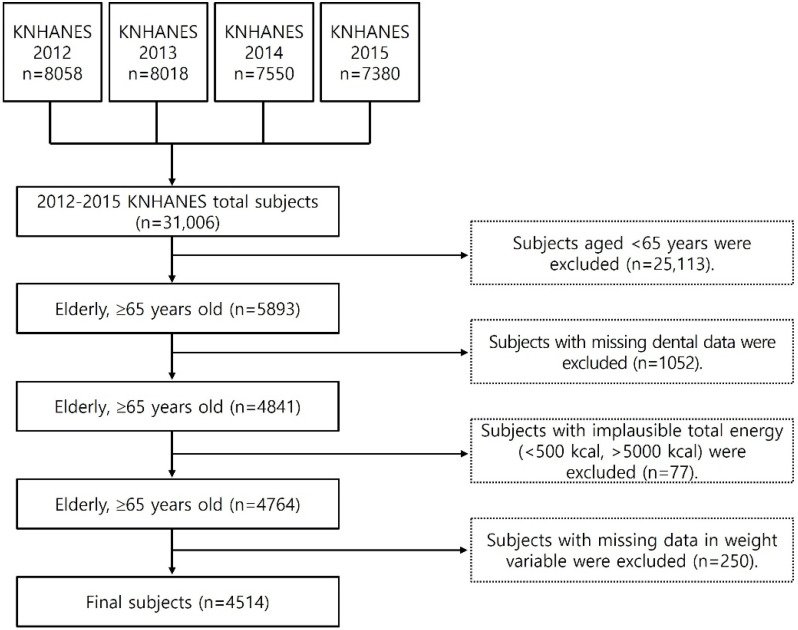
The flow chart for subject samples of this study.

This study selected elderly people over 65 years old who participated in the nutritional survey and health interview of the KNHANES 2012–2015. To examine the intake of plant food (fruit and non-starchy vegetable) of these subjects, adults who were 65 y of age or older who participated in the 24 h recall method were first selected as subjects (5893 persons). Some of the subjects who consumed less than 500 kcal or more than 5000 kcal (77 persons) were excluded. Outlier data on subjects who did not participate in the dietary survey (24 h recall survey) were also excluded (250 persons). A total of 4514 people were selected in the study (Figure 1). The KNHANES data used in this study were approved by the KDCA Institutional Review Board (IRB approval numbers: 2012-01EXP-01-2C, 20N-03-4C, and 2013-12EXP-03-5C). Among these, the KNHANES was exempt from review regarding research ethics based on the Bioethics and Safety Act from 2015 to 2017 [25].

### 2.3. Definition of Periodontal Disease

Periodontal examination was performed using the community periodontal index (CPI) certified by the WHO [26] as the criterion for the classification of PD in the 5th and 6th KNHANES [11,27]. The KNHANES oral examination was performed by two public health dentists affiliated with the disease control headquarters and 30 public health dentists supported by a city and a rural area. The oral cavity was divided into six sites: upper right posterior, upper anterior, upper left posterior, lower right posterior, lower anterior, and lower left posterior. The probing depth was measured at six sites around each index tooth, and the highest score was recorded.

Six segments were evaluated for each mouth. Upper right second molar (#17), upper right first molar (#16), upper right central incisor (#11), upper left first molar (#26), upper left second molar (#27), lower right second molar (#47), lower right first molar (#46), lower left central incisor (#31), lower left first molar (#36), and lower left second molar (#37) were designated as index teeth. The CPI scores range from 0 to 4 and are defined as: 0, healthy; 1, bleeding on gentle probing; 2, dental calculus and bleeding; 3, shallow pockets of 4 or 5 mm; and 4, deep periodontal pockets of 6 mm or more. The lower the value, the better the periodontal condition [26]. The highest CPI score was 4 points, which was based on the cases where two or more measurable residual teeth per segment were present.

A previous study reported that a CPI score of 3 or 4 indicates clinically significant periodontitis [28]. As with that previously reported definition of periodontitis [29], this study defines PD as a CPI score of 3 in more than one of six sextants and severe PD as a CPI score of 3 in all of six sextants.

### 2.4. Covariates

General characteristics of the subjects, such as gender, age, marital status, education level, residential area, and occupation, were analyzed. Among these characteristics, age was classified as 65–74 years or ≥75 years, and marital status was classified as unmarried or married. The education level was classified as less than high school graduate (<12 years), high school graduate (12 years), or college degree or higher (>12 years). Residential area was classified as city area or rural area, and occupational status was classified as occupied or unoccupied. Finally, household income level was classified using the household income level variables in KNHANES.

### 2.5. Health Behavior

For health behavior and weight status, the parameters of smoking, alcohol consumption, stress, exercise, and body mass index (BMI) were analyzed. Smoking status was divided into three groups: current smokers, ex-smokers, and nonsmokers. Alcohol consumption status was divided into more than four times a week, 2–3 times a week, 1–4 times a month, and less than once a month. The stress status was categorized into “feel it very much”, “feel it a lot”, “feel a little”, and “feel it rarely”. Exercise was classified into <1 day/week, 1–2 days/week, 3–4 days/week, and ≥5 days/week. The BMI variable used for weight status was based on the Asia-Pacific Obesity Criterion.

### 2.6. Dietary Behavior

Dietary behaviors were analyzed for breakfast, snack, location of daily meal, and eating-out frequency. Food insecurity is a dietary survey questionnaire that has been included since the 2005 KNHANES, and the question, “Which of the following best represents your family’s eating habits over the past year?” was selected based on previous studies [30,31]. Food security is defined as, “All of our family could eat enough food and a variety of foods as much as they wanted”, and mild food insecurity is defined as, “All of our family could eat enough food but couldn’t eat various kinds of food”. Moderate/severe food insecurity is defined as, “there was insufficient food sometimes or often because it was economically difficult”, and these definitions were used for analysis.

### 2.7. Food and Nutrient Intake

Food intake was categorized into 17 food groups (cereals and grain products; potatoes and starches; sugars and sweets; legumes and their products; seeds and nuts; vegetables; mushrooms; fruits; seaweeds; eggs; fish and shellfish; milk and dairy products; oils and fats; beverages; seasonings; other food; and finally meat, poultry, and their products) using food classification code (variable name: n_kindg1, n_kindg2) and food intake (variable name: nf_intk) in the food (24 h recall) data; individual intake was obtained for each survey subject. In addition, energy, macronutrient (carbohydrate, protein, and fat), and micronutrient (Ca, P, Fe, Na, K, vitamin A, carotene, retinol, thiamine, riboflavin, niacin, vitamin C) intake and contribution ratios were calculated using the daily intake for each nutrient.

### 2.8. Plant Food Intake (Fruits and Non-Starchy Vegetables)

Data on the intake of fruits or non-starchy vegetables among plant foods were obtained to calculate the total intake of fruits or/and non-starchy vegetables per day using the food classification code (variable names: n_fcode2 and n_fcode3) and food intake (variable name: nf_intk) variables among the variables of the dietary intake survey (24 h recall method) of the KNHANES. Among fruits and non-starchy vegetables, based on previous studies [32], non-starchy vegetables excluded salted vegetable and vegetable juice and fruits excluded sugar, jam, and fruit juice. Plant food intake was defined according to plant food intake standards of the World Health Organization (WHO) [33] and World Cancer Research Fund (WCRF) [34] with recommended consumption of over 400 g/day of fruits and unsalted/non-starchy vegetables.

### 2.9. Statistical Analysis

Because the KNHANES data are based on stratified multistage probability sampling rather than simple random sampling data, this statistical analysis included the weight, stratification variable (KSTRATA), and colony variable (PSU: primary sampling unit). All analyses were conducted using SAS ver. 9.4 (Statistical Analysis System, SAS Institute, Cary, NC, USA). Frequency analysis was conducted to determine the general matters related to the prevalence of periodontitis and the matters related to eating habits to show a percentage (weighted %) considering the frequency and weight. Furthermore, the intake of food and nutrients according to the prevalence of periodontitis was represented as mean and standard error using the SURVEYMEANS procedure. The significance test for this analysis was performed using SURVEYREG. A *t*-test and a general linear model were conducted for unadjusted and adjusted cases, respectively, and gender, age, and energy intake were used as adjusted variables. Finally, plant food intake was categorized into three stages (fruit, non-starchy vegetables, and fruits + non-starchy vegetables). A logistic regression analysis through SURVEYLOGISTIC was conducted to determine the relationship between the prevalence of periodontitis and the groups (T1, T2, and T3). In the groups, their intake was divided by the third quartile, and the odds ratio (OR) and the 95% confidence interval (CI) were presented. Furthermore, the influence of the variables, such as gender, age, energy intake, sodium intake, smoking, alcohol consumption, exercise frequency, stress perception, dairy intake, frequency of eating out, snack intake, food stability, and breakfast intake, was stepwise adjusted in performing multiple logistic regression analysis before conducting analysis. The p for trend value for evaluating the tendency according to the tertile was calculated using SURVEYLOGISTIC by setting the median of the third quartile as a continuous variable

## 3. Results

### 3.1. Characteristics of Subjects

Table 1 shows the general characteristics of the subjects. Of the total 4514 subjects, among whom there were more women than men in the normal group, but there was no significant difference in the periodontal group (*p* < 0.0001). The average age was 72.15 years in the normal group and 72.06 years in the periodontal group, indicating similar age distribution. In terms of the education level and income, the proportions of those who were less educated than high school graduates and those with low income were higher in the periodontal group than in the normal group. In terms of occupation, the proportion of employed individuals was significantly higher by about 6.43% in the periodontal group than in the normal group (*p* = 0.0003). In terms of region, the proportion of city dwellers was higher than that of rural area dwellers (*p* = 0.0233).

### 3.2. Health Behavior According to Prevalence of Periodontal Disease

Table 2 shows health-related factors according to the prevalence of periodontitis among survey subjects. Of the total 4514 subjects, the proportion of current smokers was significantly higher in the periodontal group (13.48%) than in the normal group (7.35%) (*p* < 0.001). In terms of alcohol consumption, the proportion of those drinking ≥4 times a week was by approximately 2.4% higher in the periodontal group (9.78%) than in the normal group (7.38%) (*p* < 0.001), and regarding stress, the proportion of those who “feel it very much” was lower in the periodontal group than in the normal group (*p* < 0.05). Regarding the parameters of exercise and weight status, there was no difference between normal and periodontal groups, with the two groups showing similar ratios.

### 3.3. Dietary Behavior According to Prevalence of Periodontal Disease

Table 3 shows the dietary behavior of subjects according to the presence of PD. The proportions of breakfast and eating-out frequency were similar in both groups, while the proportion of snack was approximately 6% lower in the periodontal group than in the normal group (*p* = 0.0031). Regarding serving location of daily meals, the proportions of home and institution location were similar in both groups, whereas the proportion of commercial location was 4.29% lower in the periodontal group than in the normal group (*p* = 0.0317).

### 3.4. Nutrients Intake According to Prevalence of Periodontal Disease

Table 4 shows the nutrient intake of subjects according to the presence of PD. Phosphorus and potassium intakes were lower in the periodontal group than in the normal group when adjusted for gender, age, and energy intake (phosphorus, adjusted *p*-value = 0.0171; potassium, adjusted *p*-value = 0.0009), and conversely, niacin intake was higher in the periodontal group than in the normal group (adjusted *p*-value = 0.0224). Vitamin C intake was higher in the normal group than in the periodontal group, and there was a significant difference regardless of the adjusting (unadjusted *p*-value = 0.0048, adjusted *p*-value = 0.0016). There were no significant differences in the remaining nutrient intake and energy contribution of carbohydrate, protein, and fat.

### 3.5. Food Intake According to Prevalence of Periodontal Disease

Table 5 shows the food intake of subjects according to the presence of PD. There was some difference in total food intake when adjusted for gender, age, and energy intake (adjusted *p*-value = 0.0260). The intake of cereals and grain products, potatoes and starches, fruits, and milk and dairy products was significantly higher in the normal group than in the periodontal group, regardless of the adjusting (unadjusted *p*-value < 0.05, adjusted *p*-value < 0.05). There were no significant difference in other food intakes between the normal and periodontal groups.

### 3.6. Plant Food (Fruits and Non-Starchy Vegetables) Intake According to Prevalence of Periodontal Disease

Table 6 shows the plant food (fruits and non-starchy vegetables) intake according to PD. In examining the intake of fruits, non-starchy vegetables, and fruits + non-starchy vegetables in terms of prevalence of PD, the intake of fruits and fruits + non-starchy vegetables was significantly higher in the normal group than in the periodontal group, regardless of the adjusting (unadjusted *p*-value < 0.01, adjusted *p*-value < 0.01).

### 3.7. Prevalence Ratio of Periodontal Disease by Their Tertile and Intake Range of Plant Food

Table 7 shows the range of the third quartile intake of plant food (fruits + non-starchy vegetables) and the prevalence of periodontitis accordingly. Based on the plant intake classification (fruits, non-starchy vegetables, and fruits + non-starchy vegetables) shown in Table 6, these items were further divided into tertiles (T1, T2, and T3). In the case of the prevalence according to the tertile and intake range of plant food, the fruit intake was between 41.85% and 48.91%, and the prevalence according to the non-starchy vegetable intake was between 43.75% and 48.59%. Moreover, the prevalence of periodontitis according to fruits + non-starchy vegetable intake was between 43.86% and 48.50%.

### 3.8. Relationship between Prevalence of Periodontal Disease and Plant Food Intake

Table 8 shows the relationship between plant food intake and the prevalence of PD. To determine the relationship with the prevalence of periodontitis according to the intake of fruits, non-starchy vegetables, and fruits + non-starchy vegetables, a SURVEYLOGISTIC procedure was used. The results suggested a relationship with the prevalence of periodontitis with the intake of fruits and fruits + non-starchy vegetables but not with the intake of non-starchy vegetables alone. First, on studying the relationship with the periodontal group according to the third quartile of the intake of fruits, which was related to the prevalence of periodontitis, the prevalence of periodontitis tended to decrease by 24.8% (OR = 0.752) as it moved toward the T3 group (fruit intake range: 198.20–4287.50 g) based on the T1 group (fruit intake range: 0 g) in Model 1 without adjusting (*p* for trend = 0.001). In Model 2, which was adjusted for gender, age, and energy intake, the prevalence of periodontitis in the T3 group decreased by 25.4% (OR = 0.746). In Models 3, which were stepwise adjusted for smoking, alcohol consumption, stress status, weight status, intake of milk and dairy products, food security, snack, eating out frequency, breakfast, education, household income, and marital status, the prevalence of periodontitis, compared with the T1 group, was lower by 21.5% (OR = 0.785) only in the T3 group.

Furthermore, on studying the relationship with the periodontitis group according to the third quartile of fruits + non-starchy vegetable intake, which was related to the prevalence of periodontitis, the prevalence of periodontitis tended to decrease by about 25.6% (OR = 0.744) as it moved toward the T3 group (fruits + non-starchy vegetable intake range: 409.23–5899.35 g) based on the T1 group (fruits + non-starchy vegetable intake range: 0–77.13 g) in Model 1 without adjusting (*p* for trend = 0.0072). In Model 2, which was adjusted for gender, age, and energy intake, the prevalence of periodontitis in the T3 group tended to decrease by about 30.5% (OR = 0.695). In Model 3, which was additionally adjusted for alcohol consumption, stress status, and weight status, the prevalence of periodontitis, compared to the T1 group, was lower by 26.7% (OR = 0.733) only in the T3 group (*p* for trend= 0.0101).

## 4. Discussion

With the elderly population increasing globally, it is necessary to prepare for health, medical, nutritional, and food issues for the aged society. This global phenomenon has resulted from improved socioeconomic circumstances and health and medical technology development, and South Korea is not an exception. To systemically manage the increase in the elderly population, the United Nations (UN) classifies societies according to the proportion of elderly people: aging society (more than 7%), aged society (more than 14%), and super-aged or post-aged society (more than 20%). By 2026, it is forecasted that elderly people will make up 20.8% of South Korean society, making it a super-aged society in which one in every five people is elderly [35].

This rapid aging of the population has raised social concerns such as health issues, the social and economic burden of elderly support, the prolongation of healthy lives, and improvement of quality of life [36]. Furthermore, health promotion via improved dietary habits in the elderly, particularly in dental health management, is becoming more significant [37]. This has resulted in increased concerns for elderly oral health care [38], and studies on elderly oral health and nutritional status have been conducted [37,39]. Some of the previous studies have reported that nutritional imbalance occurs from weakened masticatory function when the number of teeth decreases below 20 [37]. Another study reported that dental caries and PD are closely related to food intake and nutritional status, further influencing masticatory function [40,41].

PD is caused by and progresses by complex risk factors [42]. In this study, it was found that PD in the elderly is related not only to the food group intakes but also to other health-related factors such as smoking, alcohol consumption, and stress, as well as dietary behaviors such as snacking habits and serving location. Furthermore, several studies have been conducted on relationships between socioeconomic factors and PD. Although it was not significant, this study also showed that the lower the educational and income levels of elderly people, the higher their risk of PD. Therefore, further in-depth study analyzing the relationships between these factors is required. Analyses of general KNHANES data also revealed higher PD risks with lower educational and income levels [43], which was consistent with the result of a study by Sabba et al. that revealed insufficient dental disease care in those with lower education levels [44].

This study investigated the relationships between PD prevalence and various dietary-related factors, such as the breakfast frequency, snacking habits, serving location, and frequency of dining out. Among these factors, the frequency of snacking was negatively correlated with PD prevalence. A previous study suggested that elderly people with snacking habits have better nutritional status, which may explain this phenomenon [45,46]. In regard to the serving location, PD prevalence was lower when a commercial location was frequently used. Whether this is due to the influence of the food and nutrition intake provided by different serving locations should be further studied.

According to previous studies, PD and obesity have a close relationship. Analyses of South Korean adults who participated in the 2013 KNHANES revealed that the odd ratio of PD in overweight subjects with a high BMI was 1.68 times higher than that in normal-weight subjects, and those with high waist circumference had a 1.37 times higher odds ratio (OR) than those with a normal waist circumference, showing a positive correlation with obesity [47]. Furthermore, PD decreased with a balanced healthy diet and physically active lifestyle, revealing a significant association between obesity and PD [48]. However, other studies on adult subjects using the KNHANES have not found any significance of BMI in PD occurrence and have only indicated the relationship between waist circumference and PD [49]. This result is consistent with a study that used the NHANES in America, which showed that waist–hip ratio (WHR) had a higher association with PD than BMI did. However, PD prevalence and BMI did not have any significant association in this study. A further study that includes detailed obesity classifications (mild, moderate, and morbid obesity) is required.

In previously discussed nutritional factor effects on PD, it has been reported that calcium [17], protein [20], folic acid [50], vitamin D [19], and vitamin C [51] are mainly related to PD. A study of a 12-month nutritional intervention in a group of female PD subjects showed reduced depth of the periodontal pocket and gingival index [52], indicating the relationship between nutritional status and oral health.

Among the food groups, the consumption of milk and dairy products significantly lowered periodontitis prevalence in elderly people. This result is consistent with that of previous research using KNHANES data, which has shown milk and dairy product consumption to decrease the prevalence of PD [43]. Furthermore, other studies on foreign elderly people have indicated reduced PD risks in those who consume dairy products [9,53]. Further research on the influence of fruit and vegetable consumption as well as dairy product intake on PD prevalence in the elderly is required.

In this study, the amount of fruit and non-starchy vegetable intake of South Korean elderly people was 364.15 g, which is approximately 36 g less than 400 g, which is the recommended intake by the World Health Organization (WHO) and World Cancer Research Fund (WCRF) [33,34]. The result of logistic regression analysis on the relationship between fruit and vegetable consumption and PD prevalence showed a significant result. In particular, in the unadjusted model (Model 1), which divided the fruit consumption amount into three levels, T3 (the recommended amount) had 24.8% (OR = 0.75) reduced prevalence when compared with T1 (no fruit consumption). In Model 3, in which smoking, alcohol consumption, stress obesity, energy intake, gender, and age were fully adjusted, the T3 group had a 22.5% (OR = 0.85) decrease in prevalence compared to the T1 group. When the plant-based food (fresh fruit and non-starchy vegetable) intake recommended by the WHO/WCRF was divided into three models according to adjustment levels, there was a significant difference between Model 1 (no adjustment) and Model 3 (full adjustment for smoking, alcohol consumption, stress obesity, energy intake, gender, and age) in relation to the PD prevalence (p for trend < 0.05). In Model 1, the prevalence risk in T3 was reduced by 25.6% when compared to the T1 group (OR = 0.744), and in Model 3, T3 showed a 26.7% decrease in prevalence risk compared with the T1 group (OR = 0.733).

In non-starchy vegetable consumption, none of the three subdivided groups showed any significance regarding PD prevalence, which indicates that fruit consumption was the main factor. However, when fruits and vegetables were consumed together, the OR was reduced when compared with only fruit consumption. The combined consumption of vegetables and fruits is considered to be an important factor that reduces PD, which supports the plant-based diet (fresh fruits and non-starchy vegetables) recommendation from the WHO/WCRF [33,34].

The examination of the results of previous studies and this study shows that increased fruit intake and combined vegetable and fruit intake are effective in reducing PD risks in South Korean elderly. Further studies, such as research on diets for disease prevention and intervention, clinical research on fruit and vegetable ingredients that influence disease control, research on the causal relationships between nutrients and food groups and PD, randomized clinical trials, and cohort studies are required. Furthermore, from the public health viewpoint, the significance of fruit and vegetable consumption needs to be emphasized not only to the elderly population but also to younger generations to prevent and manage PD, and diet education and public health policies need to be established accordingly.

There are several limitations to this study, First, the results cannot prove a causal relationship between plant-based food (fruit and non-starchy vegetable) intake and PD because the study was designed cross-sectionally. Additionally, it used only 24 h recall data, which makes it difficult to determine the general daily food intake due to the information sampled only covering one day [54]. Furthermore, as nutrients are consumed in the form of food, there is a limitation in investigating the relationship between periodontitis and individual nutrients.

Next, the prevalence of PD varies depending on the different diagnosis criteria, which have different standards such as probing pocket depth and clinical attachment loss. This study has the limitation of using Community Periodontal Index (CPI)-diagnosed PD, which only considers probing pocket depth. Despite these limitations, this study is significant in that it investigated a national sample size, which enabled multivariate analysis and stratification analysis by providing information on potential confounding factors. Furthermore, the KNHANES, which represents South Korean standards, was used to investigate the relationship between periodontitis in South Korean elderly and plant-based food (fruits and non-starchy vegetables) consumption. In future studies, human application tests or long-term follow-up cohort studies are required to clarify the effects of fruit and vegetable consumption on PD from a long-term perspective.

## 5. Conclusions

This study aims to investigate the relationship between the intake of plant foods (fruit + starch-free vegetables) and the prevalence of periodontal disease in elderly people 65 years of age or older as part of a study on the dietary lives of the elderly. A total of 4514 subjects over the age of 65 participated in dental surveys, health behavior interviews, and 24 h diet recall tests. As a result, it was shown that the consumption of plant foods was inversely related to the prevalence of periodontal disease in the elderly in Korea. The prevalence of periodontal disease decreased with the increasing consumption of plant foods. T3 (fruit + non-starchy vegetable intake range: 409.23–5899.35 g) showed an approximately 26.7% (OR = 0.733) reduction in PD in Model 3 compared with the T1 (0–23.7 g) criterion. In conclusion, this suggests that plant food (fruits and non-starchy vegetables) consumption should be recommended in order to prevent periodontal disease in elderly people. Further research should be performed to reveal the mechanism behind the effects of plant food (fruits and non-starchy vegetables) on periodontal disease. Randomized clinical trials and cohort studies should also be conducted to confirm the effects of plant food (fruits and non-starchy vegetables) on periodontal disease. It will be necessary to establish nutrition education and policies.

## Figures and Tables

**Table 1:** General characteristics of subjects.

Variables	Prevalence of Periodontal Disease				Total *n* = 4514		*p*-Value ^(2)^
	Normal *n* = 2476		Periodontitis *n* = 2038				
	*n*	% ^(1)^	*n*	%	*n*	%	
Total subjects	2476	54.02	2038	45.98	4514	100.0	-
Prevalence of Periodontal Disease							-
Normal	2476	100.0	-	-	2476	54.02	
Mild Periodontitis	-	-	2002	98.41	2002	45.25	
Severe Periodontitis	-	-	36	1.59	36	0.73	
Gender							<0.0001
Men	956	38.93	983	49.91	1939	43.98	
Women	1520	61.07	1055	50.09	2575	56.02	
Age							0.3246
65~74y	1628	66.66	1367	68.30	2995	67.41	
75y+	848	33.34	671	31.70	1519	32.59	
Average (Mean ± S.E.)	72.15 ± 0.12		72.06 ± 0.13		72.11 ± 0.10		<0.0001^(3)^
Marital status							0.2817
Married	2459	99.59	2030	99.77	4489	99.67	
Single	10	0.41	7	0.23	17	0.33	
Education Level							0.1488
Less than graduate of high school	1618	71.47	1383	73.64	3001	72.48	
Graduate of high school	393	17.34	315	17.32	708	17.33	
College or higher	253	11.19	184	9.04	437	10.20	
Region							0.0233
City	1843	76.35	1390	71.54	3233	74.14	
Rural area	633	23.65	648	28.46	1281	25.86	
Job status							0.0003
Occupied	683	28.97	699	35.40	1382	31.95	
Unoccupied	1584	71.03	1185	64.60	2769	68.05	
Household income							0.2375
Low	1128	45.48	988	48.67	2116	46.94	
Middle-low	694	27.72	546	27.21	1240	27.49	
Middle-high	364	15.49	291	14.53	655	15.05	
High	266	11.31	191	9.60	457	10.53	

^(1)^ Weighted %, ^(2)^ *p*-value by chi-square, ^(3)^ *p*-value by *t*-test.

**Table 2:** Factors related to health according to prevalence of periodontitis in survey subjects.

Variables	Prevalence of Periodontal Disease				Total *n* = 4514		*p*-Value ^(2)^
	Normal *n* = 2476		Periodontitis *n* = 2038				
	*n*	% ^(1)^	*n*	%	*n*	%	
Smoking status							<0.0001
Current smoking	173	7.35	246	13.48	419	10.17	
Ex-smoking	600	27.05	575	31.73	1175	29.21	
Nonsmoking	1521	65.61	1074	54.79	2595	60.62	
Drink status							<0.0001
<1 time a month	1521	65.28	1167	60.27	2688	62.97	
1~4 times a month	448	20.12	360	18.62	808	19.43	
2~3 times a week	164	7.22	195	11.33	359	9.12	
≧4 times a week	165	7.38	181	9.78	346	8.49	
Stress status							0.0220
Feel it very much	90	4.01	63	2.90	153	3.50	
Feel a lot	362	16.85	263	13.79	625	15.44	
Feel a little	1137	49.65	997	52.04	2134	50.75	
Rarely	702	29.49	567	31.27	1269	30.31	
Exercise							0.3597
<1 day/week	1779	78.68	1512	80.43	3291	79.48	
1~2 days/week	158	6.24	94	4.90	252	5.62	
3~4 days/week	123	5.48	91	4.83	214	5.18	
≧5 days/week	201	9.60	179	9.84	380	9.71	
Weight status							0.4485
Underweight (BMI < 18.5 kg/m^2^)	90	3.55	59	2.86	149	3.23	
Normal (18.5 kg/m^2^ ≤ BMI < 23 kg/m^2^)	889	35.91	695	34.28	1584	35.16	
Overweight (23 kg/m^2^ ≤ BMI < 25 kg/m^2^)	626	24.74	540	26.30	1166	25.46	
Obesity (BMI ≥ 25 kg/m^2^)	867	35.80	742	36.57	1609	36.15	

^(1)^ Weighted %, ^(2)^ *p*-value by chi-square.

**Table 3:** Dietary behavior according to prevalence of periodontitis in survey subjects.

Variables	Prevalence of Periodontal Disease				Total *n* = 4514		*p*-Value ^(2)^
	Normal *n* = 2476		Periodontitis *n* = 2038				
	*n*	% ^(1)^	*n*	%	*n*	%	
Breakfast							0.7950
5~7 times a week	1603	92.83	1414	92.77	3017	92.80	
3~4 times a week	52	2.92	34	2.36	86	2.65	
1~2 times a week	17	1.32	25	1.54	42	1.43	
Rarely (<1/week)	47	2.93	45	3.33	92	3.12	
Snack							0.0031
No	1385	57.45	1245	63.24	2630	60.11	
Yes	1091	42.55	793	36.76	1884	39.89	
Home							0.1584
Not eating	696	29.61	632	32.68	1328	31.02	
Eating	1780	70.39	1406	67.32	3186	68.98	
Commercial location							0.0317
Not eating	1211	50.21	1088	54.50	2299	52.18	
Eating	1265	49.79	950	45.50	2215	47.82	
Institution location							0.5239
Not eating	2355	94.78	1936	95.28	4291	95.01	
Eating	121	5.22	102	4.72	223	4.99	
Eating-out Frequency							0.2879
≧1 times a day	88	4.00	94	4.70	182	4.32	
5~6 times a week	125	5.32	94	4.73	219	5.05	
3~4 times a week	165	6.46	144	6.90	309	6.66	
1~2 times a week	635	25.75	462	22.69	1097	24.34	
1~3 times a month	807	31.95	693	34.47	1500	33.11	
Rarely	651	26.53	546	26.52	1197	26.53	

^(1)^ Weighted %, ^(2)^ *p*-value by chi-square.

**Table 4:** Nutrient intake according to prevalence of periodontitis in survey subjects.

		Prevalence of Periodontal Disease				Total *n* = 4514		Unadjusted *p*-Value ^(1)^	Adjusted *p*-Value ^(2)^
		Normal *n* = 2476	Periodontitis *n* = 2038						
		Mean	SE	Mean	SE	Mean	SE		
Energy (kcal)		1670.95	17.00	1711.92	19.29	1689.92	13.80	0.3652	0.3090
Carbohydrate (g)		299.51	3.08	302.38	3.50	300.84	2.51	0.7033	0.9933
Protein (g)		53.96	0.70	54.83	0.77	54.36	0.55	0.5186	0.5330
Fat (g)		24.68	0.51	25.08	0.58	24.86	0.41	0.8512	0.2313
Ca (mg)		416.63	8.49	412.27	8.51	414.61	6.22	0.6632	0.2339
P (mg)		910.49	12.02	905.01	12.03	907.95	9.01	0.6499	0.0171
Fe (mg)		15.22	0.27	15.93	0.56	15.55	0.30	0.4369	0.5671
Na (mg)		3302.92	62.33	3461.29	65.40	3376.23	48.25	0.2240	0.6046
K (mg)		2721.24	46.77	2614.11	41.69	2671.66	34.11	0.0598	0.0009
Vitamin A (μg, RE)		657.86	41.13	619.63	24.36	640.17	25.02	0.8864	0.9330
Carotene (μg)		3516.70	244.70	3318.73	141.60	3425.07	148.14	0.6854	0.8677
Retinol (μg)		59.67	5.28	55.04	4.27	57.53	3.45	0.5606	0.5300
Thiamine (mg)		1.53	0.02	1.58	0.03	1.55	0.02	0.9829	0.1550
Riboflavin (mg)		0.99	0.02	0.97	0.02	0.98	0.01	0.2323	0.1419
Niacin (mg)		12.81	0.20	12.89	0.20	12.85	0.15	0.8458	0.0224
Vitamin C (mg)		101.50	3.62	90.32	3.08	96.33	2.65	0.0048	0.0016
**Energy contribution**									
Carbohydrate (%)		74.60	0.24	74.70	0.28	74.65	0.19	0.7251	0.3592
Protein (%)		12.75	0.08	12.72	0.11	12.73	0.07	0.7411	0.8234
Fat (%)		12.66	0.18	12.58	0.21	12.62	0.15	0.5241	0.2784

^(1)^*p*-value by *t*-test, ^(2)^ Adjusted for gender, age and energy intake using proc surveyreg of SAS.

**Table 5:** Food intake according to prevalence of periodontitis in survey subjects.

g/Day	Prevalence of Periodontal Disease				Total *n* = 4514		Unadjusted *p*-Value ^(1)^	Adjusted *p*-Value ^(2)^
	Normal *n* = 2476		Periodontitis *n* = 2038					
	Mean	SE	Mean	SE	Mean	SE		
Cereals and grain products	221.79	6.94	219.36	7.86	220.67	6.26	0.0340	0.0109
Potatoes and starches	33.09	2.93	22.34	2.27	28.11	2.08	0.0014	0.0016
Sugars and sweets	5.68	0.37	5.81	0.44	5.74	0.30	0.8494	0.6580
Legumes and their products	31.96	1.77	28.83	1.83	30.51	1.41	0.8585	0.8686
Seeds and nuts	4.56	0.54	4.49	0.68	4.53	0.44	0.5436	0.7444
Vegetable ^(3)^	239.83	9.35	238.91	9.81	239.40	7.91	0.4650	0.0908
Mushrooms	2.56	0.59	2.65	0.40	2.60	0.38	0.5127	0.4527
Fruits ^(4)^	128.94	6.66	106.42	6.08	118.51	5.05	0.0013	0.0009
Seaweeds	14.24	2.03	12.62	1.80	13.49	1.50	0.4364	0.4415
Meat, poultry and their products	38.96	2.68	42.97	3.26	40.82	2.22	0.8934	0.8421
Eggs	9.10	0.63	7.91	0.64	8.55	0.49	0.2202	0.1571
Fishes and shell fishes	51.17	3.71	53.80	4.29	52.39	3.35	0.0543	0.0793
Milks and dairy products	45.68	2.80	34.74	2.73	40.61	2.10	0.0160	0.0234
Oils and fats	3.26	0.16	3.46	0.21	3.35	0.15	0.1122	0.2533
Beverages	61.38	3.95	73.77	5.24	67.12	3.43	0.0666	0.3604
Seasonings	20.22	0.99	19.44	0.93	19.86	0.75	0.2071	0.0906
Other food	0.35	0.14	0.36	0.13	0.35	0.10	0.4217	0.6232

^(1)^*p*-value by *t*-test, ^(2)^ Adjusted for gender, age and energy intake, ^(3)^ Including salted vegetable, kimchi and vegetable juice, ^(4)^ Including fruits preserved in sugar, jam and fruits juice.

**Table 6:** Plant food intake according to prevalence of periodontitis in survey subjects.

g/Day	Prevalence of Periodontal Disease				Total *n* = 4514		Unadjusted *p*-Value ^(1)^	Adjusted *p*-Value ^(2)(3)^
	Normal *n* = 2476		Periodontitis *n* = 2038					
	Mean	SE	Mean	SE	Mean	SE		
Fruits ^(4)^	124.15	6.44	103.11	6.03	114.40	4.94	0.0013	0.0009
Non-starchy vegetable ^(5)^	149.50	6.97	143.94	6.63	146.92	5.56	0.1587	0.0519
Fruits^(4)^ + Non-starchy vegetable ^(5)^	273.65	11.05	247.05	11.00	261.33	9.11	0.0006	<0.001

^(1)^*p*-value by *t*-test, ^(2)^*p*-value by GLM (Generalized Linear Model), ^(3)^ Adjusted for gender, age and energy intake, ^(4)^ Excluding fruits preserved in sugar, jam and fruits juice. ^(5)^ Excluding salted vegetable and vegetable juice.

**Table 7:** Prevalence ratio of periodontal disease by their tertile and intake range of plant food.

Variables	T1		T2		T3		Total	
	Mean	SE	Mean	SE	Mean	SE	Mean	SE
Fruits	*n* = 1709		*n* = 1403		*n* = 1402		*n* = 4514	
Mean	0		96.04	1.88	447.15	0.78	166.17	5.18
Median	0		95.38	3.30	351.76	9.84	74.06	5.29
Intake range	0		0.08–197.1		198.20–4287.50		0–4287.50	
Prevalence of periodontal disease (*n*, Weighted %)								
Normal	884	51.09	777	53.59	815	58.01	2476	54.02
Periodontitis	825	48.91	626	46.41	587	41.85	2038	45.98
Non-starchy vegetable	*n* = 1504		*n* = 1505		*n* = 1505		*n* = 4514	
Mean	39.72	0.84	145.02	1.04	402.96	7.76	197.98	4.38
Median	38.96	1.41	141.84	1.26	330.64	6.01	143.28	3.29
Intake range	0–87.79		87.82–211.52		211.64–5899.35		0–5899.35	
Prevalence of periodontal disease (*n*, Weighted %)								
Normal	837	54.42	795	51.41	844	56.25	2476	54.02
Periodontitis	667	45.58	710	48.59	661	43.75	2038	45.98
Fruits + Non-starchy vegetables	*n* = 1504		*n* = 1505		*n* = 1505		*n* = 4514	
Mean	85.07	1.74	280.38	1.97	733.80	11.45	364.15	7.17
Median	84.55	3.32	272.42	2.71	619.83	9.36	270.80	5.84
Intake range	0–77.13		77.92–409.20		409.23–5899.35		0–5899.35	
Prevalence of periodontal disease (*n*, Weighted %)								
Normal	802	51.50	819	54.48	855	56.14	2476	54.02
Periodontitis	702	48.50	686	45.52	650	43.86	2038	45.98

**Table 8:** Relationship between prevalence of periodontal disease and plant food intake.

	Model 1 ^(1)^	Model 2 ^(2)^	Model 3 ^(3)^	Model 4 ^(4)^
Fruits				
T1	1 (Reference)	1	1	1
T2	0.905 (0.639–1.013) ^(6)^	0.918 (0.752–1.121)	0.927 (0.761–1.130)	0.963 (0.790–1.173)
T3	0.752 (0.631–0.896)	0.746 (0.612–0.908)	0.785 (0.645–0.956)	0.864 (0.700–1.697)
*p* for trend ^(5)^	0.0010 (-)	0.0015 (-)	0.0037 (-)	0.1640 (-)
Non-starchy vegetables				
T1	1	1	1	1
T2	0.886 (0.722–1.087)	1.021 (0.828–1.260)	1.057 (0.856–1.304)	1.064 (0.858–1.319)
T3	1.061 (0.866–1.300)	0.835 (0.672–1.036)	0.873 (0.700–1.090)	0.904 (0.721–1.133)
*p* for trend ^(5)^	0.2161 (-)	0.0907 (-)	0.2111 (-)	0.3523 (-)
Fruits+Non-starchy vegetables				
T1	1	1	1	1
T2	0.874 (0.709–1.079)	0.837 (0.675–1.039)	0.841 (0.678–1.045)	0.902 (0.721–1.128)
T3	0.744 (0.599–0.924)	0.695 (0.549–0.881)	0.733 (0.579–0.929)	0.796 (0.619–1.024)
*p* for trend ^(5)^	0.0072 (-)	0.0026 (-)	0.0101 (-)	0.0745 (-)

^(1)^ Model 1: Unadjusted, ^(2)^ Model 2: Adjusted for gender, age and energy intake, ^(3)^ Model 3: Adjusted for gender, age, energy intake, smoking, drink, stress and weight status, ^(4)^ Model 4: Adjusted for gender, age, energy intake, smoking, drink, stress status, intake of milk and dairy products, food security, snack, eating-out frequency, breakfast, education level, household income, and marital status. ^(5)^ *p* for trends were obtained by Surveylogistic Procedure of SAS. ^(6)^ Odds ratio (95% CI: confidence interval).

## Data Availability

All data were obtained from the Korea Disease Control and Prevention Agency and are available with the permission of the Korea Disease Control and Prevention Agency. The data in this study were from the Korea National Health and Nutrition Examination Survey.

## Abbreviations

BMI, Body Mass Index; CPI, Community periodontal index; KDCA, Korea Disease Control and Prevention Agency; KNHANES, the Korea National Health and Nutrition Survey; OR, Odd ratio; PD, periodontal disease; WCRF, World Cancer Research Fund; WHO, World Health Organization

## References

[1] Pihlstrom B.L., Michalowicz B.S., Johnson N.W. (2005). Periodontal diseases. Lancet.

[2] Burt B. (2005). Position paper: Epidemiology of periodontal diseases. J. Periodontol..

[3] Kornman K.S. (2008). Mapping the pathogenesis of periodontitis: A new look. J. Periodontol..

[4] Nunn M.E. (2003). Understanding the etiology of periodontitis: An overview of periodontal risk factors. Periodontology.

[5] Mealey B.L., Oates T.W. (2006). Diabetes mellitus and periodontal disease. Periodontology.

[6] Preshaw P.M., Alba A.L., Herrera D., Jepsen S., Konstantinidis A., Makrilakis K., Taylor R. (2012). Periodontitis and diabetes: A two-way relationship. Diabetologia.

[7] Kim C.W., Park J.W., Suh J.Y., Cho J.Y., Lee J.M. (2009). The expressions of C-reactive protein and macrophage colony-stimulating factor in gingival tissue of human chronic periodontitis with hypertension. J. Korean Acad. Periodontol..

[8] Linden G.J., Linden K., Yarnell J., Evans A., Kee F., Patterson C.C. (2012). All-cause mortality and periodontitis in 60–70-year-old men: A prospective cohort study. J. Clin. Periodontol..

[9] Shimazaki Y., Shirota T., Uchida K., Yonemoto K., Kiyohara Y., Iida M., Saito T., Yamashita Y. (2008). Intake of dairy products and periodontal disease: The Hisayama Study. J. Periodontol..

[10] GBD 2015 Disease and Injury Incidence and Prevalence Collaborators (2016). Global, regional, and national incidence, prevalence, and years lived with disability for 310 diseases and injuries, 1990–2015: A systematic analysis for the Global Burden of Disease Study 2015. Lancet.

[11] Ministry of Health and Welfare, Korea Disease Control and Prevention Agency (2016). The Sixth Korea National Health and Nutrition Examination Survey (KNHANES VI), 2013–2015.

[12] Al-Zahrani M.S. (2006). Increased intake of dairy products is related to lower periodontitis prevalence. J. Periodontol..

[13] Najeeb S., Zafar M.S., Khurshid Z., Zohaib S., Almas K. (2016). The Role of Nutrition in Periodontal Health: An Update. Nutrients.

[14] Schierle R.E. (2005). Nutrition and Periodontal Disease. Dent. Clin. N. Am..

[15] Choi J. (2008). Relation between Food Intake and Self-Recognition of Major Oral Disease on the Korean Adults. Master’s Thesis.

[16] Palacios C., Joshipura K.J., Willett W.C. (2009). Nutrition and health: Guidelines for dental practitioners. Oral Dis..

[17] Nishida M., Grossi S.G., Dunford R.G., Ho A.W., Trevisan M., Genco R.J. (2000). Calcium and the risk for periodontal disease. J. Periodontol..

[18] Nishida M., Grossi S.G., Dunford R.G., Ho A.W., Trevisan M., Genco R.J. (2000). Dietary vitamin C and the risk for periodontal disease. J. Periodontol..

[19] Garcia M.N., Hildebolt C.F., Miley D.D., Dixon D.A., Couture R.A., Spearie C.L., Langenwalter E.M., Shannon W.D., Deych E., Mueller C. (2011). One-year effects of vitamin D and calcium supplementation on chronic periodontitis. J. Periodontol..

[20] Enwonwu C.O. (1995). Interface of malnutrition and periodontal diseases. Am. J. Clin. Nutr..

[21] Park Y.W. (2009). Bioactive Components in Milk and Dairy Products.

[22] Koo S.M., Park Y.J., Hwang J.Y. (2013). Association between consumption of fruits and vitamin c and generalized periodontitis in Korean adults: The 2007–2010 Korean National Health and Nutrition Examination Surveys. J. Korean Soc. Dent. Mater..

[23] Oh K., Lee J., Lee B., Kweon S., Lee Y., Kim Y. (2007). Plan and Operation of the 4th Korea National Health and Nutrition Examination Survey (KNHANES IV). Korean J. Epidemiol..

[24] The Homepage of Korea National Health and Nutrition Examination Survey. http://kosis.kr/.

[25] Korea Centers for Disease Control and Prevention (2018). Guideline for Seventh Korea National Health and Nutrition Examination Survey (KNHANES VII).

[26] World Health Organization (1997). Oral Health Surveys: Basic Methods.

[27] Ministry of Health and Welfare, Korea Centers for Disease Control and Prevention (2016). The 5th Korea National Health and Nutrition Examination Survey (KNHANES V), 2010–2012.

[28] Inagaki K., Kurosu Y., Yoshinari N., Noguchi T., Krall E.A., Garcia R.I. (2005). Efficacy of periodontal disease and tooth loss to screen for low bone mineral density in Japanese women. Calcif. Tissue Int..

[29] Lee K., Kim J. (2019). Dairy Food Consumption is Inversely Associated with the Prevalence of Periodontal Disease in Korean Adults. Nutrients.

[30] Shim J.S., Oh K.W., Nam C.M. (2008). Association of Household Food Security with Dietary Intake; Based on the Third (2005) Korea National Health and Nutrition Examination Survey (KNHANES III). J. Nutr. Health.

[31] Lee S.J., Lee K.W., Oh J.E., Cho M.S. (2015). Nutritional and health consequences are associated with food insecurity among Korean elderly: Based on the fifth (2010) Korea National Health and Nutrition Examination Survey (KNHANES V-1). J. Nutr. Health.

[32] Kwon J.H., Shim J.E., Park M.K., Paik H.Y. (2009). ‘Evaluation of fruits and vegetables intake for prevention of chronic disease in Korean adults aaged 30 years and over’ using the Third Korea National Health and Nutrition Examination Survey (KNHANES III) 2005. Korean J. Nutr..

[33] World Health Organization. http://www.who.int/elena/titles/fruit_vegetables_ncds/en/.

[34] World Cancer Research Fund & American Institute for Cancer Research (2007). Food, Nutrition, Physical Activity, 499 and the Prevention of Cancer: A Global Perspective.

[35] Statistics Korea (2011). Population Projections for Korea: 2010–2060.

[36] Kim T.M., Lee S.G., Jeon S.Y. (2006). The relations of social support to the health behaviors and health status in the elderly. Korean J. Health Educ. Promot..

[37] Han S.Y., Kim C.S. (2016). Does denture-wearing status in edentulous South Korean elderly persons affect their nutritional intakes. Gerodontology.

[38] Petersen P.E., Yamamoto T. (2005). Improving the oral health of older people: The approach of the WHO global oral health programme. Community Dent. Oral Epidemiol..

[39] Luis D., Guzman A.L. (2006). Nutritional status of adult patients admitted to internal medicine departments in public hospitals in Castilla y Leon, Spain—A multi-center study. Eur. J. Intern. Med..

[40] Palmer C.A. (2001). Important relationships between diet, nutrition, and oral health. Nutr. Clin. Care.

[41] Henshaw M.M., Calabrese J.M. (2001). Oral health and nutrition in the elderly. Nutr. Clin. Care.

[42] Van Dyke T.E., Dave S. (2005). Risk factors for periodontitis. J. Int. Acad. Periodontol..

[43] Koo S.M., Seo D.G., Park Y.J., Hwang J.Y. (2014). Association between consumption of milk and dairy products, calcium and riboflavin, and periodontitis in Korean adults: Using the 2007–2010 Korea. J. Nutr. Health.

[44] Sabbah W., Tsakos G., Chandola T., Sheiham A., Watt R.G. (2007). Social Gradients in Oral and General Health. J. Dent. Res..

[45] Kerver J.M., Yang E.J., Obayashi S., Bianchi L., Song W.O. (2006). Meal and snack patterns are associated with dietary intake of energy and nutrients in US adults. J. Am. Diet. Assoc..

[46] Walls A.W.G., Steele J.G. (2004). The relationship between oral health and nutrition in older people. Mech. Ageing Dev..

[47] Lee Y.K., Park J.R. (2013). The relationship of obesity and periodontal disease by age. J. Korean Soc. Dent. Hyg..

[48] Han D.H., Lim S.Y., Sun B.C., Paek D.M., Kim H.D. (2010). Visceral fat area-defined obesity and periodontitis among Koreans. J. Clin. Periodontol..

[49] Kim E.J., Jin B.H., Bae K.H. (2011). Periodontitis and obesity: A study of the fourth Korean national health and nutrition examination survey. J. Periodontol..

[50] Yu Y.-H., Kuo H.-K., Lai Y.-L. (2007). The association between serum folate levels and periodontal disease in older adults: Data from the National Health and Nutrition Examination Survey 2001/02. J. Am. Geriatr. Soc..

[51] Zhang M.F., Chen R.J., Tang W., Zhang H.F. (2012). The relationship between dietary factors and susceptibility of periodontal disease. Shanghai J. Stomatol..

[52] Jenxsch A., Eick S., Rassoul F., Purschwitz R., Jentsch H. (2009). Nutritional intervention in patients with periodontal disease: Clinical, immunological and microbiological variables during 12 months. Br. J. Nutr..

[53] Adegboye A.R., Christensen L.B., Holm-Pedersen P., Avlund K., Boucher B.J., Heitmann B.L. (2012). Intake of dairy products in relation to periodontitis in older Danish adults. Nutrients.

[54] Lee J., Shin A. (2015). Vegetable and fruit intake in one-person household: The Korean National Health and Nutrition Examination Survey (2010–2012). J. Nutr. Health.

